# HNRNPC mediates lymphatic metastasis of cervical cancer through m6A-dependent alternative splicing of FOXM1

**DOI:** 10.1038/s41419-024-07108-4

**Published:** 2024-10-07

**Authors:** Yun-Yun Liu, Meng Xia, Zhi-Bo Chen, Yuan-Dong Liao, Chun-Yu Zhang, Li Yuan, Yu-Wen Pan, Hua Huang, Huai-Wu Lu, Shu-Zhong Yao

**Affiliations:** 1 Department of Obstetrics and Gynecology, The First Affiliated Hospital of Sun Yat-sen University, Guangzhou, Guangdong China; 2 Guangdong Provincial Clinical Research Center for Obstetrical and Gynecological Diseases, Guangzhou, Guangdong China; 3 Department of Gynecological Oncology, Sun Yat-sen Memorial Hospital of Sun Yat-Sen University, Guangzhou, Guangdong China; 4 Guangdong Provincial Key Laboratory of Malignant Tumor Epigenetics and Gene Regulation, Sun Yat-Sen Memorial Hospital, Sun Yat-Sen University, Guangzhou, Guangdong China; 5 Department of Cardiovascular Surgery, Sun Yat-sen Memorial Hospital, Sun Yat-sen University, Guangzhou, Guangdong China

**Keywords:** Metastasis, Gynaecological cancer

## Abstract

Cervical cancer (CCa) patients with lymph node (LN) metastasis face poor prognoses and have limited treatment options. Aberrant N6-methyladenosine (m^6^A) modification of RNAs are known to promote tumor metastasis, but their role in CCa remains unclear. Our study reveals that HNRNPC, an alternative splicing (AS) factor and m^6^A reader, increases tumor-related variants through m^6^A-dependent manner, thereby promoting lymphatic metastasis in CCa. We found that HNRNPC overexpression correlates with lymphatic metastasis and poorer prognoses in CCa patients. Functionally, knocking down HNRNPC markedly inhibited the migration and invasion of several CCa cell lines, while supplementing HNRNPC restored the malignant phenotypes of these cells. Mechanistically, HNRNPC regulates exon skipping of FOXM1 by binding to its m6A-modified motif. Mutating the m^6^A site on FOXM1 weakened the interaction between HNRNPC and FOXM1 pre-RNA, leading to a reduction in the metastasis-related FOXM1-S variant. In conclusion, our findings demonstrate that m^6^A-dependent alternative splicing mediated by HNRNPC is essential for lymphatic metastasis in CCa, potentially providing novel clinical markers and therapeutic strategies for patients with advanced CCa.

## Introduction

Cervical cancer (CCa) is the fourth most common cancer among women worldwide and the leading cause of death from gynecologic malignancies [1]. Patients with early-stage CCa have a favorable 5-year survival rate exceeding 90% with comprehensive treatment, including surgery and adjuvant therapy [2]. However, the occurrence of lymph node metastasis (LNM) significantly increases recurrence and mortality rates [3], making it a major cause of treatment failure and patient death [4]. Nevertheless, the underlying mechanism of lymphatic metastasis in CCa remains unclear. N6-methyladenosine (m^6^A) is the most prevalent RNA modification and plays a significant role in post-transcriptional regulation [5]. It has been found to promote tumor metastasis in various cancers [6]. Heterogeneous nuclear ribonucleoprotein C (HNRNPC), a recently identified m^6^A reader [7], is crucial in regulating precursor RNA (pre-RNA), particularly in the alternative splicing (AS) of pre-mRNA [8]. Accumulating studies have shown that high HNRNPC expression is associated with poor prognosis in several tumors [9–11]. However, its role in gynecological oncology has not been well explored. Our investigation demonstrates that HNRNPC is greatly upregulated in lymph node-positive CCa tissues and is associated with inferior overall survival (OS) and progression-free survival (PFS). Functionally, HNRNPC invigorates lymphatic metastasis of CCa both in vivo and in vitro. Mechanistically, HNRNPC binds to Forkhead box protein M1 (FOXM1) pre-mRNA in an m^6^A-dependent manner, driving the AS of FOXM1, leading to the high expression of the pro-metastatic variant FOXM1-S. Our study reveals a significant and novel mechanism where HNRNPC, as an AS factor and m^6^A reader, facilitates lymphatic metastasis in CCa, offering new insights into potential therapeutic targets.

## Materials and methods

### Human tissue samples

We recruited ten CCa patients with tumors smaller than 2 cm, diagnosed at Sun Yat-sen Memorial Hospital (Table S1). Tumor and adjacent normal tissues were collected and stored in RNA later at −80 °C. Additionally, sixty-one CCa patients diagnosed with FIGO (The International Federation of Gynecology and Obstetrics) IB1-IIIC1 and seventeen patients with benign uterus or ovarian diseases who underwent total hysterectomy were enrolled (Table S1). CCa tumor tissues and normal cervix samples were collected and preserved in RNA later at −80 °C. The formula “${\rm{N}}=\frac{{Z}_{\alpha }^{2}P(1-P)}{{\delta }^{2}}$” (*α* = 0.05, *P* = 48%, *δ* = 0.1) was used to estimate the sample size of patients for immunohistochemistry (IHC) staining. Ninety-two CCa patients and ninety-six CCa patients, diagnosed via histology and who underwent radical hysterectomy plus pelvic lymphadenectomy at two independent gynecology oncology centers (the First Affiliated Hospital and Sun Yat-sen Memorial Hospital of Sun Yat-sen University), were included for IHC staining. Inclusion criteria included patients with preoperative biopsy pathology confirmed as squamous, adenocarcinoma, or adenosquamous carcinoma, proposed for surgical treatment, free of syphilis, human immunodeficiency virus, and other immune-related diseases, and no history of oral immunosuppressive drugs. The informed consent was obtained, and the ethical approval was granted by the Committees for Ethical Review of Research Involving Human Subjects of Sun Yat-sen University (SYSU-2021-580).

### Immunohistochemical (IHC) analysis

Tissue sections were stained with an anti-HNRNPC antibody (1:200, #11760-1, Proteintech) and Horseradish Peroxidase (HRP)-goat-anti-rabbit antibody (Zhong Shan-Golden Bridge Biological Technology). Staining intensity (negative, 0; mild, 1; moderate, 2; severe, 3) and the proportion of positive staining cells (negative, 0%; ≤25%, 1; >25% and ≤50%, 2; >50% and ≤75%, 3; >75% and ≤100%, 4) were multiplied to calculate staining scores.

### TCGA and GEO data mining

The clinical profiles and gene expression data of 306 CCa patients were obtained from https://portal.gdc.cancer.gov/. Data from CCa patients in GSE7803, GSE6791, and GSE63514 were sourced from https://www.ncbi.nlm.nih.gov/geo/.

### RNA isolation, RT-PCR, and Western Blot

Total RNA and protein were extracted following the protocol of the RNA isolation kit (Esunbio, China) and Protein isolation kit (BestBio, China). Reverse transcription and Polymerase Chain Reaction (PCR) were performed with SYBR qPCR Master Mix kits (Vazyme, China). Primers used in the experiments are listed in Table S2. Western Blot was performed as described in our previous studies.

### Cell cultures

Human cervical squamous cancer cell lines Siha, MS751, Caski, and adenocarcinoma cell lines Hela, Hela229 were purchased from the cell bank of Shanghai. Cells were cultured in DMEM or 1640 containing 10% fetal bovine serum (Gibco, America) at 37 °C and were tested for mycoplasma contamination before and during experiments.

### RNAi and lentivirus transduction

SiRNA oligonucleotides targeting HNRNPC, Wilms tumor 1 associated protein (WTAP), and negative control siRNA were purchased from GenePharma (Suzhou, China) and are listed in Table S3. SiRNA transfections were conducted according to the manufacturer’s instructions of RNAiMAX (Invitrogen, America). HNRNPC expression was knocked down by stable transduction with pcDNA3.1-puro lentivirus following our previous protocol. FOXM1-L and FOXM1-S variant plasmids, along with FOXM1-m^6^A-wild-type, and mutation plasmids were packaged into lentivirus to establish stable expression cell lines. Puromycin was used at 2 µg/ml to select stable expression cells.

### Cell metastasis assays and proliferative assays

Transwell, wound healing, and proliferative assays were performed as described in our previous studies.

### Footpad implantation and popliteal-lymph-node metastasis assay

Four-week-old female BALB/c nude mice were procured from the Experimental Animal Center of Guangdong Province and were randomly allocated to control and experimental groups. The formula “${\rm{N}}=\frac{{Z}_{{\rm{\alpha }}}^{2}P(1-P)}{{\delta }^{2}}$” (*α* = 0.05, *P* = 20%, *δ* = 0.3) was used to estimate the sample size. A total of 1 × 10^7^ cells were suspended in 50 µL of Hanks’ solution and inoculated into the footpads of the mice to build the LNM model. Blinding was used when labeling cell suspension and during inoculation. Fluorescence signals were monitored using an In Vivo Imaging System (IVIS) (PerkinElmer, USA) after 8 weeks. Popliteal lymph nodes were the regional draining lymph nodes and the first site of metastasis from footpad tumor, they were excised and fixed in 4% formalin. Immunohistochemical staining with Pan-CK and hematoxylin and eosin (HE) staining were performed. All animal experimental procedures were approved by the Institutional Animal Care and Use Committee of Sun Yat-sen University (SYSU-IACUC-2021-B1906).

### RNA immunoprecipitation (RIP) assay and RIP-qPCR analysis

For the RIP assay and subsequent qPCR analysis, 2 × 10^7^ Hela and Siha cells were lysed using RIP lysis buffer. The RIP assays were performed with the EZ-Magna RIP kit (Millipore, USA). A 5-µg amount of anti-HNRNPC antibody (#11760-1-AP, Proteintech) was used for the RIP incubation.

### m^6^A mutation assay

Hela and Siha cells were plated in six-well plates and transfected with either wild-type or m^6^A mutant (A-to-C mutation) FOXM1 vectors.

### Statistical analysis

Statistical analyses were conducted using SPSS 18.0 software. The F test was applied to estimate homogeneity of variance (*P* > 0.05 was considered to be homogeneous). The student’s *t*-test was applied to compare expression differences, and the Chi-Square test assessed the association of clinicopathological features. Kaplan–Meier survival curves and log-rank tests were employed to compare OS and PFS. A significance level of *P* < 0.05 was considered statistically significant.

## Results

### HNRNPC correlates with LN metastasis and is an unfavorable prognostic factor in CCa

**Fig. 1:**
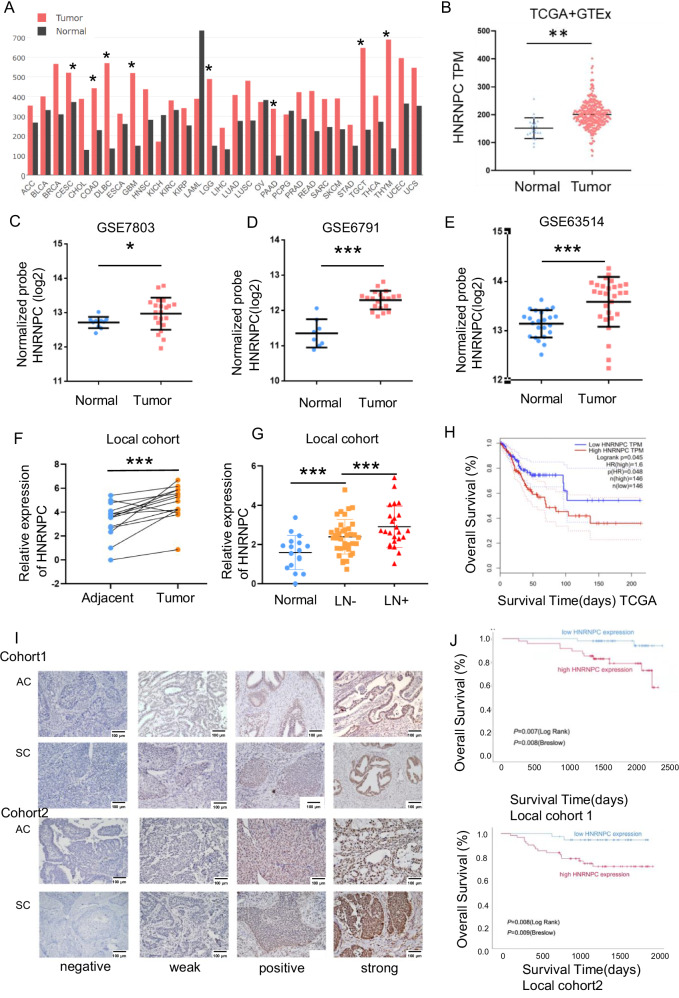
HNRNPC overexpression in cervical cancer: clinical and prognostic implications. **A** TCGA data reveals elevated HNRNPC expression in tumors across various cancer types compared to normal tissues. **B** Significantly increased HNRNPC expression observed in TCGA cervical cancers. **C**–**E** GEO database analysis confirms high HNRNPC expression in CCa tissues compared to normal tissues (GSE7803, GSE6791, GSE63514). **F** PCR analysis shows significant upregulation of HNRNPC in CCa tumors compared to adjacent tissues in a local cohort (*n* = 10). **G** Local cohort PCR data indicates higher HNRNPC levels in lymphatic metastasis CCa compared to patients without lymph node metastasis and normal cervix. **H** TCGA data analysis links high HNRNPC RNA expressions to shorter overall survival (*n* = 292, *P* value = 0.048). **I** HNRNPC expression patterns in cervical adenocarcinoma and squamous cell carcinoma from two independent gynecology departments. **J** Kaplan–Meier curves demonstrate high HNRNPC expression as an unfavorable prognostic factor for overall survival in both local cohorts (cohort1: *n* = 91, cohort2: *n* = 96) with *P* values of 0.007 and 0.008, respectively. *P < 0.05, **P < 0.01, ***P < 0.001. LN lymph node, TCGA The Cancer Genome Atlas, AC adenocarcinoma, SC squamous cell carcinoma.

Analysis of the TCGA database revealed that most m6A-related genes were differentially expressed in CCa compared to normal cervix tissues (Fig. S1A). Among these, HNRNPC expression was higher in CCa tissues than in normal cervix tissues (Fig. 1A, B). Subsequent data mining from GEO and Oncomine databases confirmed the overexpression of HNRNPC in CCa tissues (Figs. S1B, and 1C–E). PCR validation from our cohort also showed higher HNRNPC expression in tumor samples compared to adjacent normal samples (Fig. 1F). Additionally, HNRNPC expression correlated with lymph node (LN) metastatic CCa and was significantly associated with poor prognosis in CCa patients (Fig. 1G, H, Table S4). HNRNPC was overexpressed in LN-positive CCa tissues and metastatic CCa cell lines compared to LN-negative tissues and non-metastatic cell lines (Fig. S1C, D). These expression patterns suggest that HNRNPC may play a potentially pro-LN-metastasis role in CCa.

To further validate HNRNPC’s role in CCa, we conducted IHC staining on paraffin sections of CCa patients from two independent gynecology oncology surgery centers. HNRNPC expression was consistently lower in adjacent normal tissues than in tumors (Fig. S1E). Patients with high HNRNPC expression had higher LN metastasis rates (34.04% and 66.67%) compared to those with low HNRNPC expression (12.25 and 40%) in two cohorts (Tables 1, 2, Fig. S1F). Kaplan–Meier(K-M) curves indicated that HNRNPC expression was an unfavorable risk factor for OS and PFS in CCa patients (Figs. S1G, H, and 1I, J). These findings indicated the potential value of HNRNPC in LN metastasis of CCa.

**Table 1:** Clinical-pathologic information of patients in cohort 1.

Clinical-pathology information		HNRNPC expressions		*χ*^2^ value	*P* value
		Low	High		
Age	<45	17	14	45.125	0.049*
	≥45	32	33		
Pathology	Squamous cell carcinoma	43	42	0.081	0.960
	Adenocarcinoma	6	5		
Grade	High	3	0	4.286	0.117
	Medium	12	14		
	Low	34	33		
Stage	I	40	40	0.209	0.428
	II	9	7		
Lymph node status	Negative	43	31	6.629	0.01*
	Positive	6	16		
LVSI	Negative	30	28	0.979	0.613
	Positive	8	11		
Myometrial invasion	<1/2	15	15	0.023	0.989
	≥1/2	34	30		
Parametrial infiltration	Negative	48	42	4.537	0.103
	Positive	1	5		
Tumor size	≤4 cm	45	43	22.269	0.674
	>4 cm	4	3		
Lymph cell infiltration	Negative	25	22	0.17	0.418
	Positive	24	25		
Vaginal stump involvement	Negative	48	45	0.395	0.484
	Positive	1	2		

*LVSI* lympho-vascular space involvement.

*P < 0.05.

**Table 2:** Clinical-pathologic information of patients in cohort 2.

Clinical-pathology information		HNRNPC expressions		*χ*^*2*^ value	*P* value
		Low	High		
Age	<45	9	10	33.093	0.159
	≥45	26	47		
BMI	≤24	18	18	107.317	0.027*
	>24	17	39		
Pathology	Squamous cell carcinoma	29	49	162	0.452
	Adenocarcinoma	6	8		
Grade	High	1	4	3.741	0.189
	Medium	10	23		
	Low	24	30		
Stage	I	12	20	3.741	0.129
	II	2	11		
	III	21	26		
Lymph node status	Negative	21	26	16.438	0.021*
	Positive	14	31		
LVSI	Negative	11	19	0.036	0.518
	Positive	24	38		
Endometrial involvement	Negative	24	26	4.606	0.026*
	Positive	11	31		
Myometrial invasion	<1/3	5	2	3.722	0.155
	≥1/3 & <2/3	5	7		
	≥2/3	25	48		
Parametrial infiltration	Negative	34	53	0.73	0.366
	Positive	1	4		
Tumor size	≤4 cm	19	38	25.458	0.264
	>4 cm	16	19		
Lymph cell infiltration	Negative	17	20	1.632	0.144
	Positive	18	37		
Vaginal stump involvement	Negative	33	55	0.247	0.492
	Positive	2	2		

*BMI* body mass index, *LVSI* lympho-vascular space involvement.

*P < 0.05.

### HNRNPC enhanced tumor invasion and proliferation in vitro and promoted CCa lymph node metastasis in vivo

**Fig. 2:**
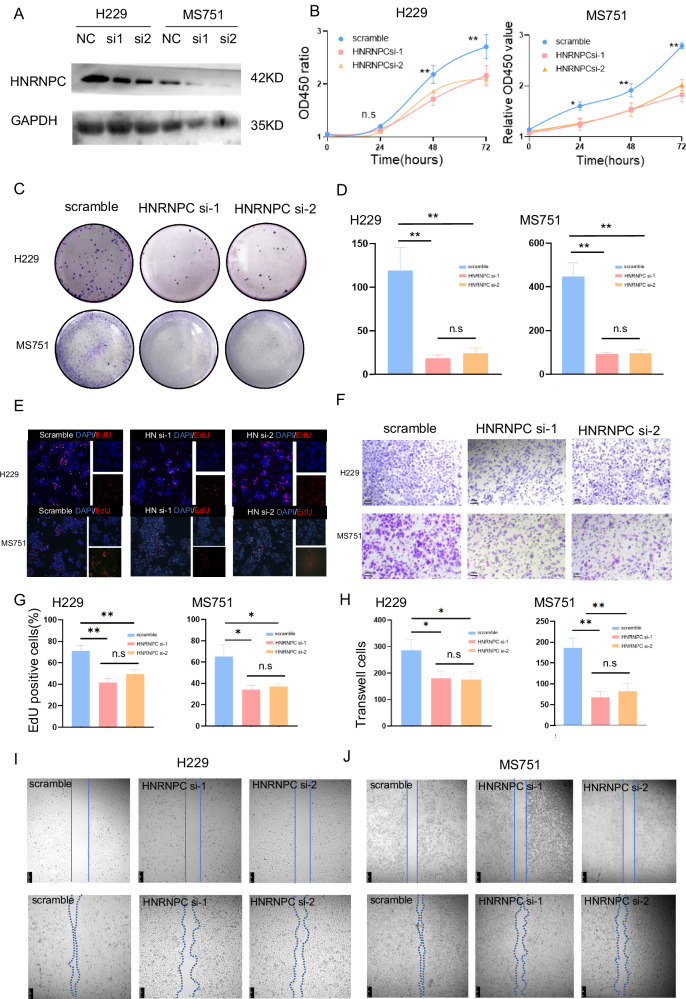
Impact of HNRNPC Downregulation on CCa Cell Proliferation, Invasion, and Migration In Vitro. **A** Western blot confirms successful downregulation of HNRNPC expression in H229 and MS751 cells using siRNAs; siRNA sequence-1 exhibits higher efficacy. **B** CCK8 assay demonstrates reduced proliferative capacity of H229 and MS751 cells upon HNRNPC downregulation (OD value = (OD_experiment_-OD_blank_)/((OD_0h_-OD_blank_), *n* = 4)). **C**, **D** Clone formation assay reveals decreased cell proliferation following HNRNPC downregulation (*n* = 3). **E**, **G** EdU assay illustrates diminished proliferation of H229 and MS751 cells after HNRNPC silencing (*n* = 3). **F**, **H** Transwell assay shows decreased invasiveness of CCa cells upon HNRNPC downregulation (*n* = 3). **I**, **J** Wound healing assay under serum-free culture conditions indicates reduced migration ability of H229 and MS751 cells 48 h after HNRNPC knocking down. *P < 0.05, **P < 0.01, n.s: *P* > 0.05. Si1 siRNA sequence-1, si2 siRNA sequence-2.

Prompted by the above findings, we examined the functions of HNRNPC in CCa cell lines by silencing its expression in Hela229 and MS751 cells (Figs. 2A, and S5). Knockdown of HNRNPC reduced invasion and migration abilities, as shown by Transwell and Wound Healing assays. Additionally, HNRNPC knockdown significantly inhibited the proliferation of CCa cell lines, as detected by clone formation, CCK-8, and EdU assays (Fig. 2B–J).

**Fig. 3:**
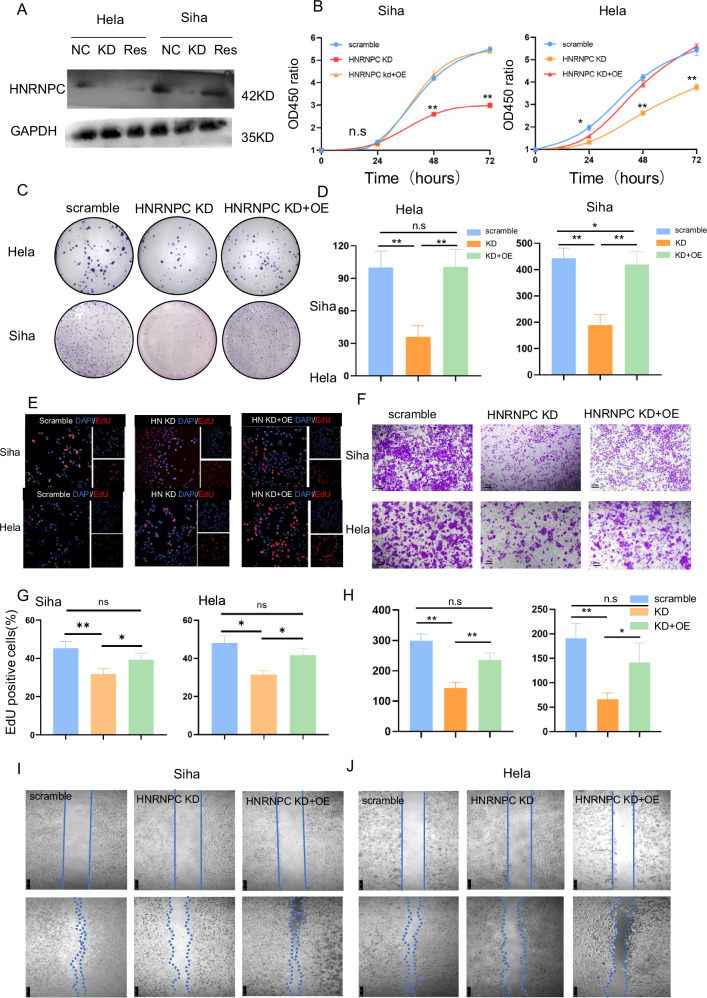
HNRNPC drives proliferation, invasion, and migration of CCa cells in vitro. **A** Western blot confirms successful rescue of HNRNPC expression in HNRNPC-silenced Hela and Siha cells through HNRNPC cDNA. **B** CCK8 assay reveals increased proliferation of Hela and Siha cells induced by HNRNPC (OD value = (OD_experiment_-OD_blank_)/((OD_0h_-OD_blank_), *n* = 4)). **C**, **D** Clone formation assay demonstrates enhanced cell proliferation upon HNRNPC rescue (*n* = 3). **E**, **G** EdU assay shows increased proliferation of Hela and Siha cells after HNRNPC rescue (*n* = 3). **F**, **H** Transwell assay indicates elevated invasiveness of CCa cells following HNRNPC rescue (*n* = 3). **I**, **J** Wound healing assay under serum-free culture conditions shows heightened migration ability of Hela and Siha cells with HNRNPC rescue. *P < 0.05, **P < 0.01, n.s:*P* > 0.05. NC negative control, KD knock down of HNRNPC, RES rescue of HNRNPC, OE over-expression of HNRNPC.

Due to the high basal expression of HNRNPC in CCa cell lines, we were unable to upregulate HNRNPC expression. In order to confirm the function of HNRNPC, we performed rescue assays in Hela and Siha cell lines (Figs. 3A, and S5). The invasion and proliferation abilities decreased in Hela and Siha after HNRNPC knockdown and were restored upon HNRNPC overexpression (Fig. 3B–J).

**Fig. 4:**
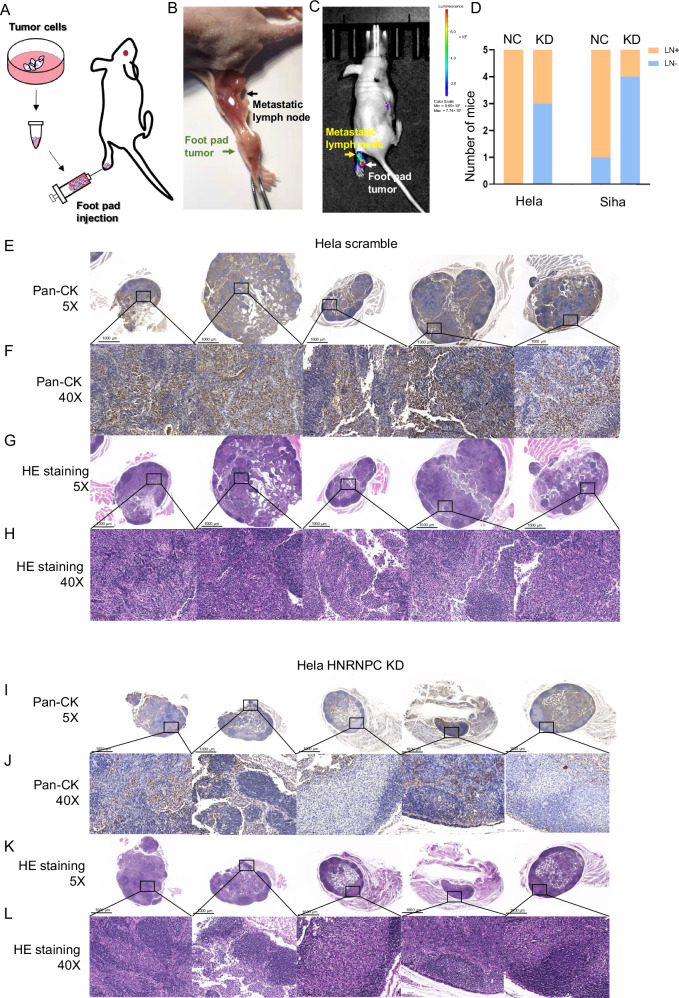
HNRNPC facilitates CCa metastasis to regional lymph nodes in vivo through the footpad-popliteal lymphatic metastasis model. **A** Diagrammatic illustration of footpad injection. **B** Anatomical atlas displaying footpad tumor and popliteal lymphatic metastasis (green arrow denotes the primary tumor on the footpad of Balb/c nude mice, black arrow indicates metastatic regional lymph nodes on the popliteal). **C** Fluorescence imaging of footpad tumor and popliteal lymphatic metastasis (white arrow signifies the primary tumor on the footpad, yellow arrow indicates metastatic regional lymph nodes on the popliteal). **D** Histogram demonstrating reduced in vivo lymph node metastasis rates in HNRNPC knock-down cells (*n* = 5). **E**, **F** Immunohistochemistry (IHC) of pan-CK staining in popliteal lymph nodes of mice injected with Hela scramble cells. **G**, **H** Hematoxylin and eosin (HE) staining of popliteal lymph nodes in mice injected with Hela scramble cells. **I**, **J** IHC of pan-CK staining in popliteal lymph nodes of mice injected with HNRNPC knocked-down Hela cells. **K**, **L** HE staining of popliteal lymph nodes in mice injected with HNRNPC knocked-down Hela cells. NC negative control, KD knock down of HNRNPC, LN negative lymph node, LN+ positive lymph node, HE hematoxylin-eosin staining.

To further determine the pro-metastasis function of HNRNPC in vivo, we created stable HNRNPC-knockdown cell lines. A CCa LNM model was constructed by transplanting HNRNPC-knockdown and negative control Hela/Siha cell lines into the footpads of nude mice (Fig. 4A–C). Two months later, we found that downregulation of HNRNPC decreased metastasis in regional draining lymph nodes (Fig. 4D), as confirmed by IHC using Pan-CK antibody (Figs. 4E–L, and S2). Taken together, these results demonstrate that HNRNPC promotes the migration and invasion of CCa cell lines both in vitro and in vivo.

### HNRNPC mediated AS of FOXM1 facilitated LN metastasis in CCa

**Fig. 5:**
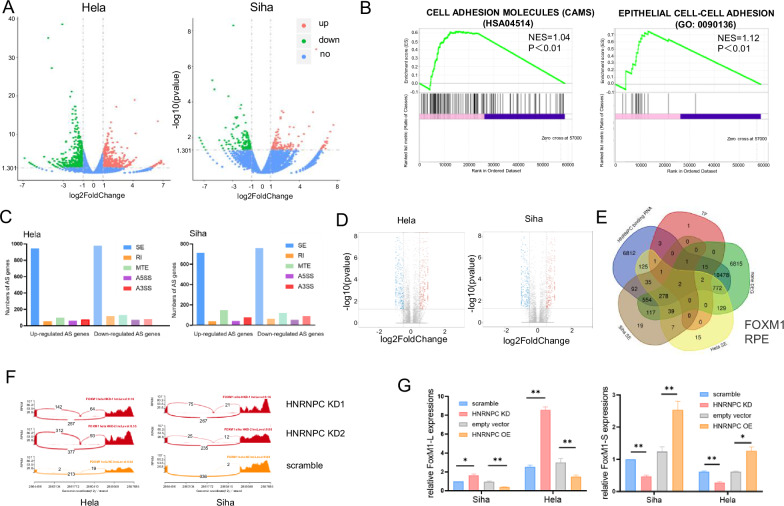
HNRNPC mediates alternative splicing of multiple molecules, including the key transcriptional factor FOXM1. **A** Volcano plots representing differentially expressed genes after HNRNPC silencing in Hela and Siha cells. **B** GSEA enrichment plots for Hela and Siha cells. **C** Histograms depicting differentially expressed alternative splicing events in Hela and Siha cells. **D** Volcano plots illustrating exon skipping events. **E** Venn diagram depicting shared alternative splicing events in Hela and Siha cells. **F** Schematic representation of skipped exons (SE) in FOXM1, showing consistent exon retention after HNRNPC knockdown in both cell lines. **G** PCR validation confirms retention of the FOXM1-L variant after HNRNPC silencing, which is reduced upon HNRNPC overexpression. *P < 0.05, **P < 0.01. KD knock down of HNRNPC, OE over expression of HNRNPC, TF transcription factor, DEG differently expressed gene, SE skipped exon, AS alternative splicing, RI retained intron, MTE mutually exclusive exons, A5SS alternative 5’ splice site, A3SS alternative 3’ splice site.

HNRNPC, an AS factor, functions exclusively in the nucleus, as confirmed by IHC staining of tumor tissues, IF staining of CCa cells, and nuclear-cytoplasmic separation experiments (Fig. S3A–C), To understand how HNRNPC promotes LN metastasis through AS regulation, RNA-seq and rMATS analysis were performed in Hela and Siha cells following HNRNPC knockdown (Fig. 5A).

Kyoto Encyclopedia of Genes and Genomes (KEGG) functional annotation of RNA-seq data indicated significant enrichment of genes in pathways such as steroid biosynthesis, transcriptional misregulation in cancer, and FoxO pathways (Fig. S4A, B). Gene Set Enrichment Analysis (GSEA) analysis indicated that cell adhesion-related-pathways were affected by HNRNPC knockdown (Fig. 5B). Skipped exon (SE) events were the most common AS events in both cell lines (Fig. 5C). HNRNPC acted as either an AS activator or a prohibitor, causing exon inclusion or skipping when HNRNPC was downregulated (Fig. 5D). To uncover how HNRNPC regulates those DEG expressions, we focused on HNRNPC-mediated AS events in transcription factors (TFs) vital to tumor metastasis. After mapping the AS sites and transcriptional levels of TFs in Hela and Siha cells, we found AS of FOXM1 and RPE were regulated in both cell lines while the total RNA levels of these two molecules were stable after HNRNPC silencing (Fig. 5E). Since FOXM1 had a profound impact on tumor progression and development, hence, we chose it for further investigation. AS analysis revealed increased VIIa exon retention of FOXM1 (FOXM1-L), with stable total RNA levels after HNRNPC depletion, validated by qPCR (Figs. 5F-G, and S4C). Consistently, restoration of HNRNPC rescued the VIIa exon skipping of FOXM1 (FOXM1-S).

**Fig. 6:**
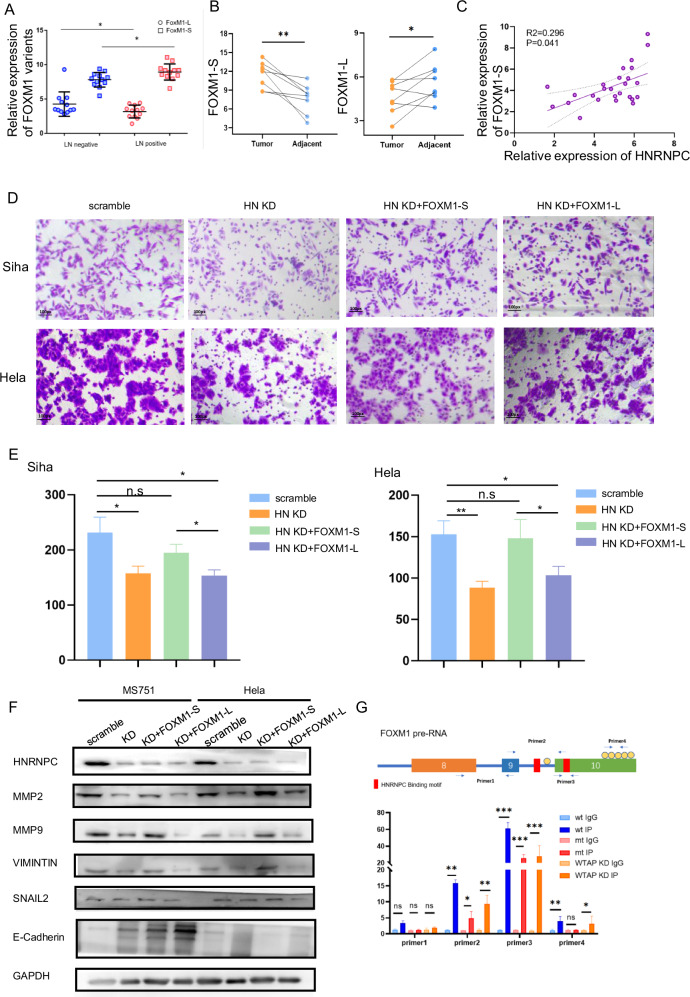
HNRNPC-Mediated Increase in FOXM1-S Variant Plays a Crucial Role in CCa Metastasis. **A** RNA levels of FOXM1-L and -S variants in cervical cancer tissues. **B** RNA levels of FOXM1-L and -S variants in paired samples of cervical cancer and adjacent tissues. **C** Regression analysis and fitting curve depicting the relationship between HNRNPC and FOXM1-S expressions in cervical cancer samples. **D**, **E** Transwell assay results indicating that the FOXM1-S variant rescues cell invasion ability, unlike the FOXM1-L variant. **F** Western blot results demonstrating elevated MMP proteins and EMT pathway molecules after complementing FOXM1-S rather than FOXM1-L. **G** RIP-PCR comparing different areas of wild-type and m6A sites mutated FOXM1. *P < 0.05, **P < 0.01, n.s *P* > 0.05. RIP RNA binding protein immunoprecipitation, wt wild type, mt m6A sites mutation, WTAP KD knocking down WTAP.

We detected the expressions of FOXM1-S and FOXM1-L by q-PCR to determine their roles in CCa (Fig. 6A, B). FOXM1-S increased in LN-metastasis CCa tissues but decreased in non-LN-metastasis tissues, while FOXM1-L showed the opposite pattern. FOXM1-S was positively correlated with HNRNPC expression (Figs. 6C, and S3D). Taken together, these results suggest HNRNPC-regulated FOXM1 variants are involved in LN metastasis of CCa. To substantiate the impact of FOXM1 isoforms and delineate the interaction between these two isoforms and HNRNPC in LN metastasis in CCa, we established FOXM1-L and FOXM1-S overexpressing lentivirus and infected them into stable HNRNPC-knockdown cell lines. Transwell assays showed that invasiveness increased with FOXM1-S overexpression, but not with FOXM1-L (Fig. 6D). Western Blot (WB) assay demonstrated that FOXM1-S overexpression rescued the expression of Epithelial-Mesenchymal Transition (EMT) pathway molecules and matrix metalloproteinase (MMP) proteins in HNRNPC-knockdown cell lines (Fig. 6F, and S5). These findings show that FOXM1-S can rescue the pro-metastasis ability of HNRNPC-deficient CCa cells by modulating invasion-associated genes.

### HNRNPC promoted the AS of FOXM1 by m^6^A modification recognition

**Fig. 7:**
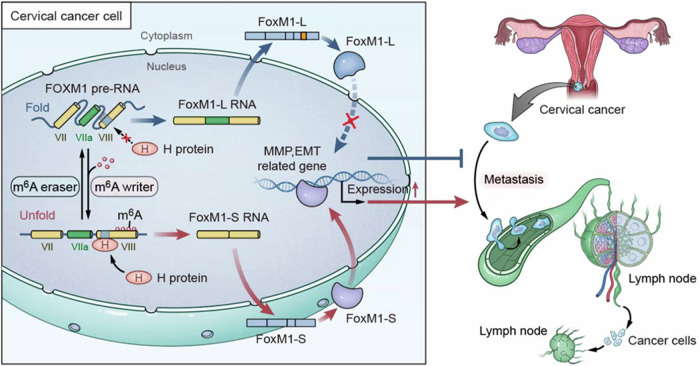
Hypothesized mechanism diagram: HNRNPC-mediated m6A-dependent binding to FOXM1 promotes lymph node metastasis in CCa through alternative splicing. In cervical cancer cells, when the VIII exon of FOXM1 undergoes m6A modification, the nuclear-located splicing factor HNRNPC recognizes and binds to the FOXM1 pre-RNA. This interaction promotes the skipping of the VIIa exon of FOXM1, leading to an elevation in the FOXM1-S variant. FOXM1-S variants are then translated into transcriptionally active FOXM1 proteins, promoting the expression of MMP and EMT-related genes, ultimately enhancing lymph node metastasis.

HNRNPC, an RNA binding protein, recognizes m^6^A modifications on pre-RNAs. To understand how HNRNPC modulates FOXM1 AS, we first investigated its binding with FOXM1 RNA. Mapping with known HNRNPC binding motifs revealed two potential binding on FOXM1 pre-RNA, one on the VIII exon and another near the VIIa exon. By prediction from websites, we found a cluster of m^6^A sites on the VIII exon, which was close to the HNRNPC binding motif. RIP-PCR assay demonstrated HNRNPC binding to the VIII exon region of FOXM1 more strongly than to IgG (Fig. 6G). To test the role of m^6^A modification in HNRNPC-mediated FOXM1 splicing, we examined HNRNPC binding to FOXM1 after m^6^A site mutation or WTAP si-RNA downregulation in Hela cells. The interaction between HNRNPC and FOXM1 was significantly suppressed after m6A site mutation or WTAP downregulation (Fig. 6G). These results further confirm that HNRNPC promotes LNM through mediating FOXM1 isoform switch in an m^6^A-dependent way(Fig. 7).

## Discussion

CCa is a prevalent and lethal malignancy worldwide [1], with LNM being a significant prognostic factor [5]. In this study, HNRNPC was found to be overexpressed in CCa tissues, especially in LN-metastasis specimens. HNRNPC’s expression level was the independent risk factor of poorer PFS, and OS. Additionally, HNRNPC promoted the invasiveness of CCa cells through in vivo and in vitro assays. Mechanistic studies uncovered the dual role of HNRNPC, acting as both a splicer and an m^6^A reader. Briefly, HNRNPC recognizes and binds to the polymeric m^6^A sites on the VIII exon of FOXM1 pre-RNA, modulating the AS of FOXM1 and enhancing the expression of FOXM1-S isoform, thereby promoting LNM of CCa.

HNRNPC is a member of the family of binding proteins for small heterogeneous nuclear RNAs, which perform biological functions in variable splicing complexes with other proteins [8]. In prostate cancer, it promotes the proliferation and migration of tumor cells and correlates with higher tumor stage, Gleason score, and poor prognosis [11]. Bioinformatics analysis of sequencing data from TCGA of glioma [10], oral squamous carcinoma [9], esophageal squamous cell carcinoma [12] shows that the expression of HNRNPC is closely related to patient prognosis, aligning with our findings in CCa. Our study found a discrepancy in LNM rates among patients with high HNRNPC expression in two independent gynecologic oncology centers. This discrepancy may be due to bias in patient sources between the centers. However, the similar findings suggest that HNRNPC has significant predictive value for LNM in CCa.

Existing studies often focus on clinicopathological parameter correlation and prognostic analysis, but further mechanistic studies on phenotypic effects of HNRNPC on tumor cells and its contribution to tumor cell metastasis and poorer prognosis have yet to be reported. A footpad implantation and popliteal lymph nodes metastasis model was used for in vivo experiment. This model aligns with the sequential metastasis pattern observed in CCa. In our future study, a pelvic LNM model will be considered. The findings in this paper suggest that high HNRNPC expression is closely associated with LNM in CCa, which may guide treatment choices. For example, CCa patients with LNM prefer radical radiotherapy over surgery [2], but preoperative pelvic Computed Tomography (CT) has limited ability to discriminate LNM. Therefore, the expression level of HNRNPC in CCa might serve as a potential biomarker for LNM and guide patient treatment. Since the chi-square test used in this study can only confirm a significant correlation between HNRNPC expression and LNM, it is a univariate test and cannot exclude the influence of other factors on this correlation. Therefore, the predictive value of HNRNPC for LNM in CCa needs further validation with larger samples.

HNRNPC has been reported as a classical alternative splicer [8]. In this study, we used sequencing to find that HNRNPC promotes exon skipping of FOXM1 in CCa cell lines, leading to an increase in FOXM1-S production and promoting LNM. Under physiological conditions, FOXM1 RNA is highly folded in three-dimensional structure, hiding HNRNPC binding sites. However, when m^6^A modification occurs in these regions, RNA accessibility increases, exposing the HNRNPC binding sites. This structural change of RNA due to m^6^A modification is known as the ‘m^6^A switch’ [13]. In pancreatic cancer [14], HNRNPC recognizes and binds to the m^6^A-modified sites on the VII exon in TATA-binding protein-associated factor 8 (TAF8) pre-RNA, promoting exon skipping of TAF8 and liver metastasis. This conclusion is supported by our data that the VIII exon of FOXM1 precursor RNA has a segment of clustered m^6^A modules, and reducing m^6^A modification in this region significantly reduces HNRNPC binding to FOXM1, hindering its AS function.

Exon skipping is the most common form of AS. In non-small cell lung cancer, 14th exon skipping in the mesenchymal-epithelial transition factor (MET) gene leads to lower ubiquitination degradation of the MET protein, resulting in sustained activation of the downstream pathway and brain metastasis [15]. In bladder cancer, the splicing factor NONO promotes exon retention of SET domain and mariner transposase fusion gene (SETMAR), increasing the formation of the short transcript SETMAR-S, and inhibiting LNM by increasing the methylation modification of the promoter of EMT-related genes [16].

FOXM1, known as an oncogene [17], has been reported as a transcriptional factor that regulates multiple pathways related to tumor metastasis and epithelial-to-mesenchymal transition (EMT) [17–19]. The 6th and 9th exons of human FOXM1 are alternative exons, resulting in at least four transcripts generated by AS [20]. In our study, FOXM1-L transcript contains the VIIa exon of FOXM1 DNA, and the protein translated from FOXM1-L may lose transcription function due to the fragment insertion [21], while the FOXM1-S transcript does not encompass the VIIa exon [22]. Currently, there are no studies on FOXM1 and LNM, or on the functional differences of FOXM1 isoforms in CCa. In this study, we found that FOXM-L had no obvious biological function in CCa, while FOXM1-S enhanced the metastatic ability of CCa cells by promoting the expression of the EMT pathway and MMPs. Overexpression of FOXM1-S enhanced the invasive ability of HNRNPC-deficient cells. The above results suggest that HNRNPC promotes exon skipping of FOXM1 precursor RNA through the m^6^A switch, leading to increased expression of FOXM1-S transcripts and promotes LNM of CCa.

## Conclusion

Our study indicated that HNRNPC is a potential and promising biomarker for LNM and prognosis in CCa patients. Mechanistically, HNRNPC mediates metastasis and proliferation by regulating AS through the “m^6^A switch” of FOXM1 pre-RNA. Based on these findings, inhibiting AS of FOXM1 may become a notable treatment decision of CCa LNM.

## Supplementary information

supplementary files

## Data Availability

The original data and informations are available to readers promptly upon request to the corresponding author.
