# Supplementary material for: HNRNPC mediates lymphatic metastasis of cervical cancer through m6A-dependent alternative splicing of FOXM1
Source: Cell Death Dis. 2024 Oct 7;15(10):732. doi: 10.1038/s41419-024-07108-4 (PMC11458786; doi:10.1038/s41419-024-07108-4)
Supplement: Supplementary file 1 — supplementary files [file 41419_2024_7108_MOESM1_ESM.pdf]

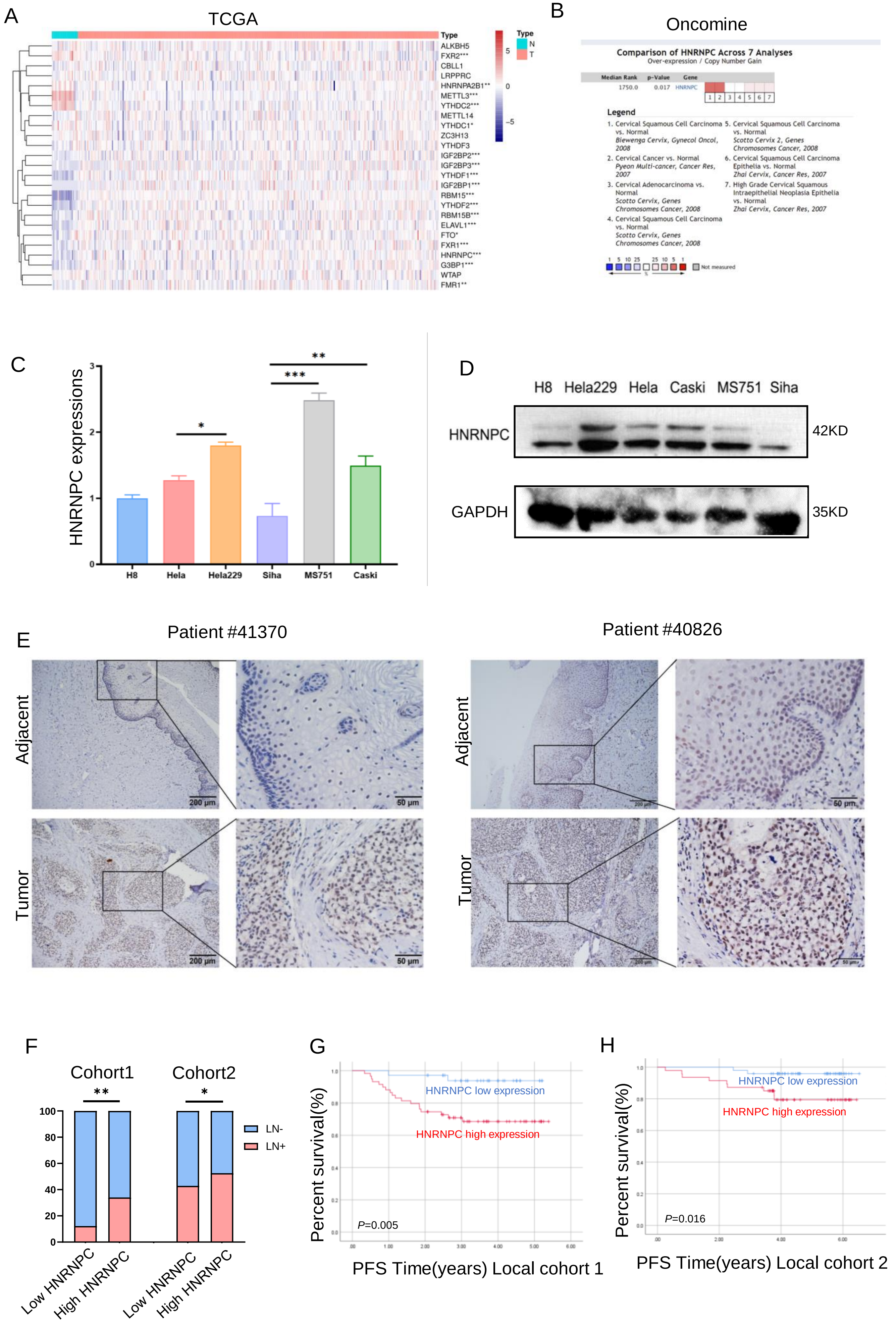

A
TCGA
Type
ALKBH5
FXR2***
CBLL1
LRPPRC
HNRNPA2B1**
METTL3***
YTHDC2***
METTL14
YTHDC1*
ZC3H13
YTHDF3
IGF2BP2***
IGF2BP3***
YTHDF1***
IGF2BP1***
RBM15***
YTHDF2***
RBM15B***
ELAVL1***
FTO*
FXR1***
HNRNPC***
G3BP1***
WTAP
FMR1**
Type
N
T
5
0
−5
B
Oncomine
Comparison of HNRNPC Across 7 Analyses
Over-expression / Copy Number Gain
Median Rank p-Value Gene
1750.0 0.017 HNRNPC
1 2 3 4 5 6 7
Legend
1. Cervical Squamous Cell Carcinoma vs. Normal Biewenga Cervix, Gynecol Oncol, 2008
2. Cervical Cancer vs. Normal Pyeon Multi-cancer, Cancer Res, 2007
3. Cervical Adenocarcinoma vs. Normal Scotto Cervix, Genes Chromosomes Cancer, 2008
4. Cervical Squamous Cell Carcinoma vs. Normal Scotto Cervix, Genes Chromosomes Cancer, 2008
5. Cervical Squamous Cell Carcinoma vs. Normal Scotto Cervix 2, Genes Chromosomes Cancer, 2008
6. Cervical Squamous Cell Carcinoma Epithelia vs. Normal Zhai Cervix, Cancer Res, 2007
7. High Grade Cervical Squamous Intraepithelial Neoplasia Epithelia vs. Normal Zhai Cervix, Cancer Res, 2007
1 5 10 25 25 10 5 1
%
Not measured
C
HNRNPC expressions
0
1
2
3
H8
Hela
Hela229
Siha
MS751
Caski
*
***
**
D
H8 Hela229 Hela Caski MS751 Siha
HNRNPC
42KD
GAPDH
35KD
E
Patient #41370
Patient #40826
Adjacent
Tumor
200 μm
50 μm
F
Cohort1
Cohort2
**
*
0
20
40
60
80
100
LN-
LN+
Low HNRNPC
High HNRNPC
Low HNRNPC
High HNRNPC
G
Percent survival(%)
HNRNPC low expression
HNRNPC high expression
P=0.005
PFS Time(years) Local cohort 1
H
Percent survival(%)
HNRNPC low expression
HNRNPC high expression
P=0.016
PFS Time(years) Local cohort 2

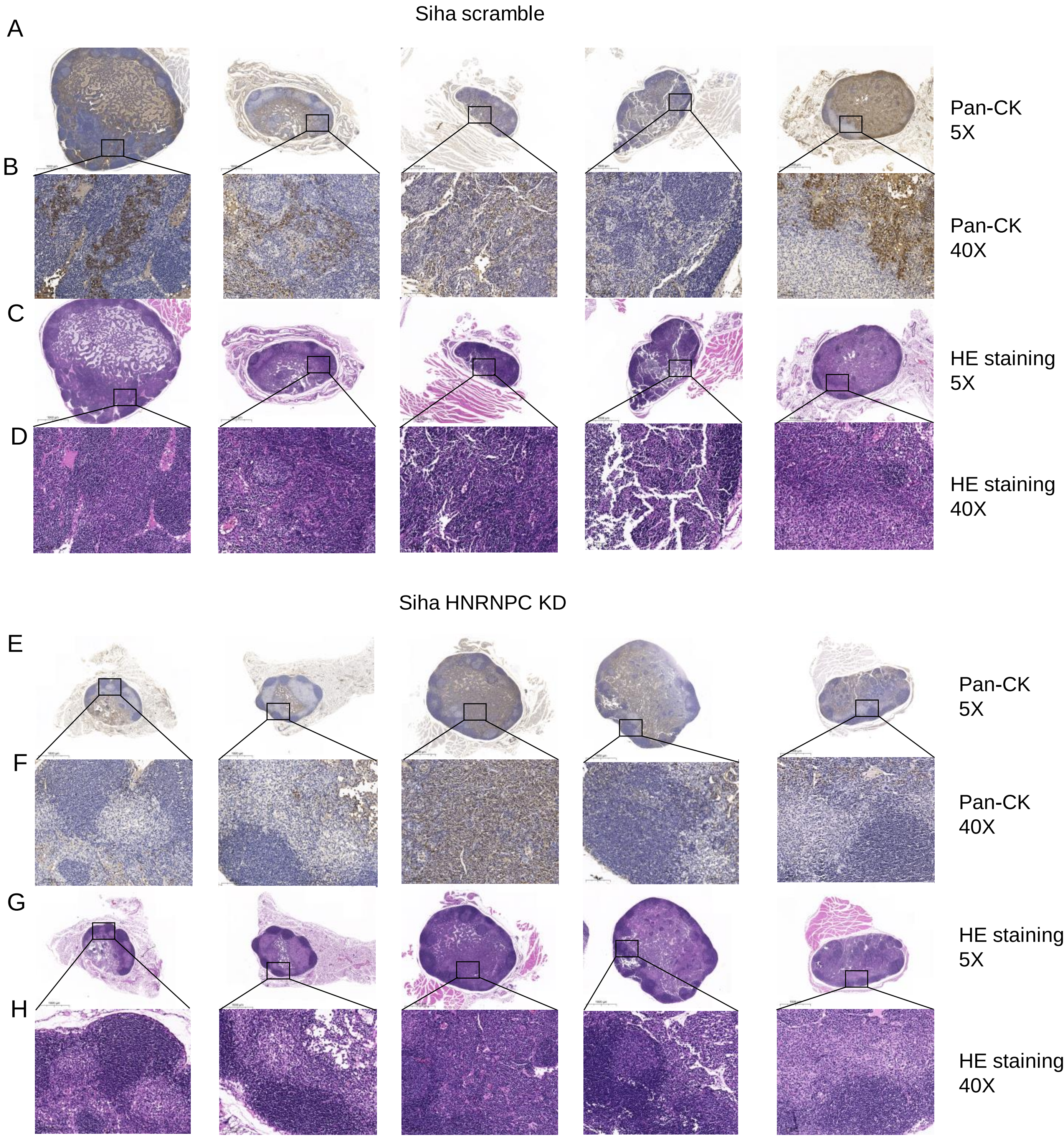
Siha scramble
A
B
C
D
Pan-CK
5X
Pan-CK
40X
HE staining
5X
HE staining
40X
Siha HNRNPC KD
E
F
G
H
Pan-CK
5X
Pan-CK
40X
HE staining
5X
HE staining
40X

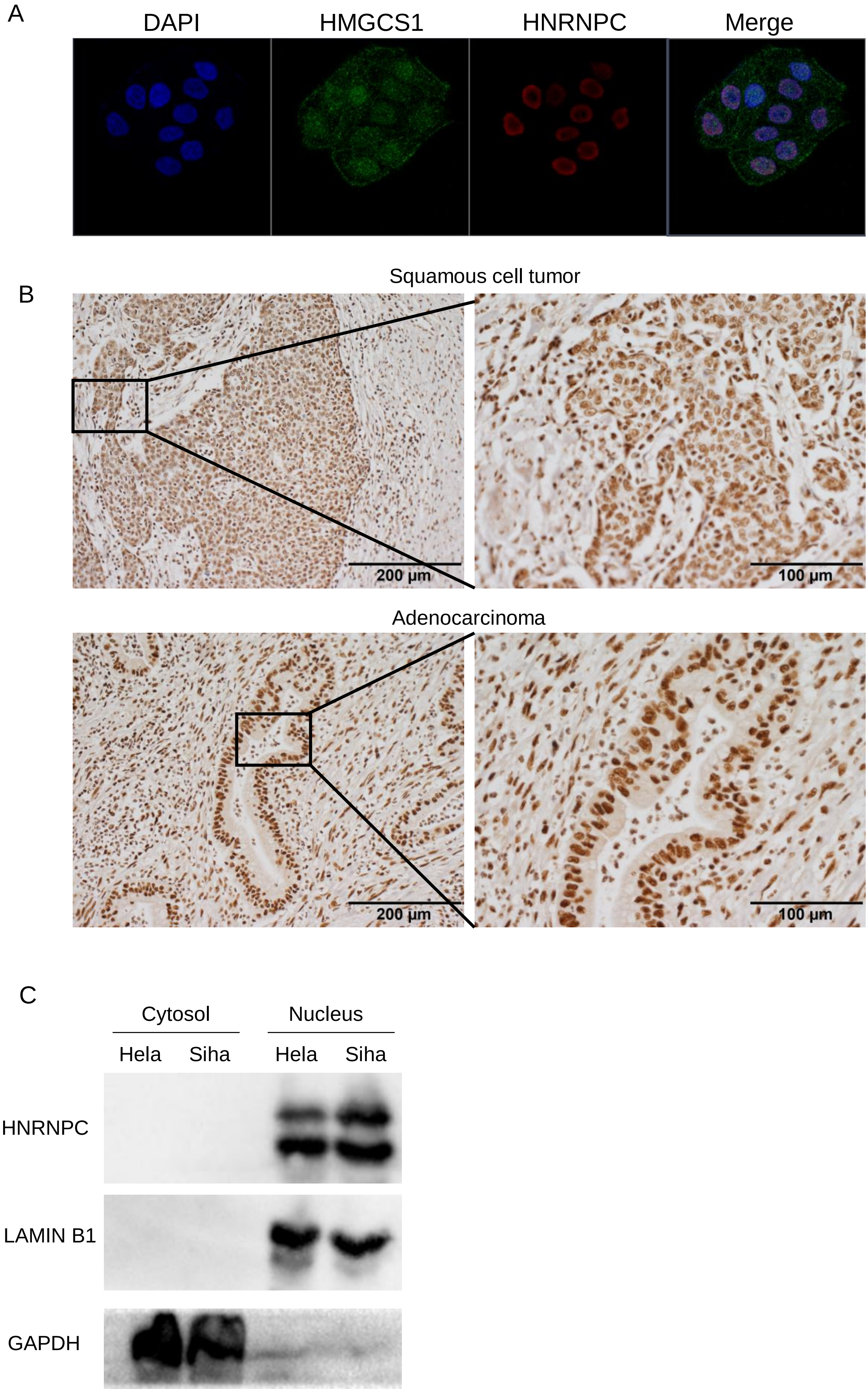

A
DAPI
HMGCS1
HNRNPC
Merge
B
Squamous cell tumor
200 μm
100 μm
Adenocarcinoma
200 μm
100 μm
C
Cytosol
Nucleus
Hela
Siha
Hela
Siha
HNRNPC
LAMIN B1
GAPDH

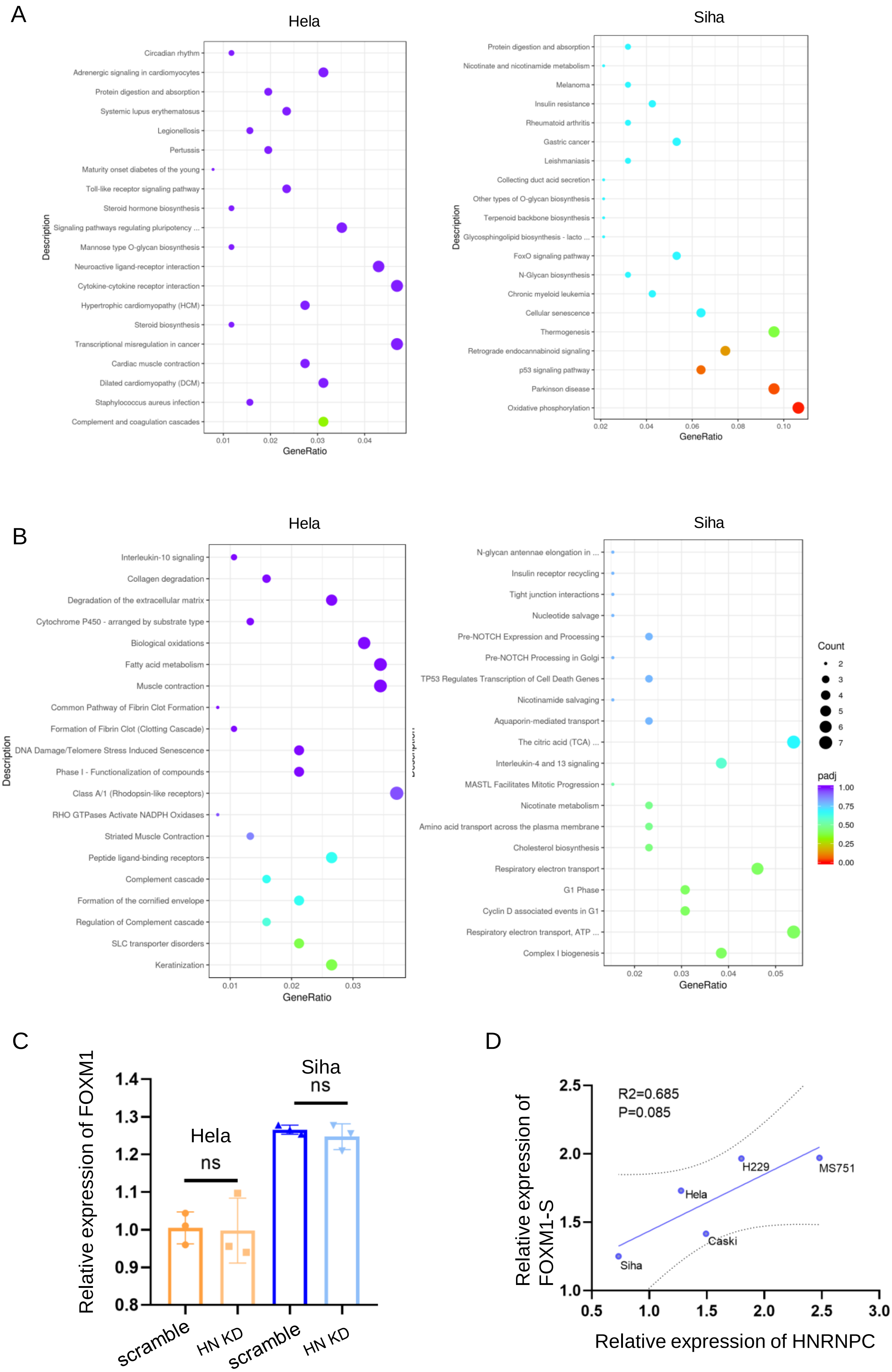

A
Hela
Circadian rhythm
Adrenergic signaling in cardiomyocytes
Protein digestion and absorption
Systemic lupus erythematosus
Legionellosis
Pertussis
Maturity onset diabetes of the young
Toll-like receptor signaling pathway
Steroid hormone biosynthesis
Signaling pathways regulating pluripotency ...
Mannose type O-glycan biosynthesis
Neuroactive ligand-receptor interaction
Cytokine-cytokine receptor interaction
Hypertrophic cardiomyopathy (HCM)
Steroid biosynthesis
Transcriptional misregulation in cancer
Cardiac muscle contraction
Dilated cardiomyopathy (DCM)
Staphylococcus aureus infection
Complement and coagulation cascades
Description
GeneRatio
Siha
Protein digestion and absorption
Nicotinate and nicotinamide metabolism
Melanoma
Insulin resistance
Rheumatoid arthritis
Gastric cancer
Leishmaniasis
Collecting duct acid secretion
Other types of O-glycan biosynthesis
Terpenoid backbone biosynthesis
Glycosphingolipid biosynthesis - lacto ...
FoxO signaling pathway
N-Glycan biosynthesis
Chronic myeloid leukemia
Cellular senescence
Thermogenesis
Retrograde endocannabinoid signaling
p53 signaling pathway
Parkinson disease
Oxidative phosphorylation
GeneRatio
B
Hela
Interleukin-10 signaling
Collagen degradation
Degradation of the extracellular matrix
Cytochrome P450 - arranged by substrate type
Biological oxidations
Fatty acid metabolism
Muscle contraction
Common Pathway of Fibrin Clot Formation
Formation of Fibrin Clot (Clotting Cascade)
DNA Damage/Telomere Stress Induced Senescence
Phase I - Functionalization of compounds
Class A/1 (Rhodopsin-like receptors)
RHO GTPases Activate NADPH Oxidases
Striated Muscle Contraction
Peptide ligand-binding receptors
Complement cascade
Formation of the cornified envelope
Regulation of Complement cascade
SLC transporter disorders
Keratinization
GeneRatio
Siha
N-glycan antennae elongation in ...
Insulin receptor recycling
Tight junction interactions
Nucleotide salvage
Pre-NOTCH Expression and Processing
Pre-NOTCH Processing in Golgi
TP53 Regulates Transcription of Cell Death Genes
Nicotinamide salvaging
Aquaporin-mediated transport
The citric acid (TCA) ...
Interleukin-4 and 13 signaling
MASTL Facilitates Mitotic Progression
Nicotinate metabolism
Amino acid transport across the plasma membrane
Cholesterol biosynthesis
Respiratory electron transport
G1 Phase
Cyclin D associated events in G1
Respiratory electron transport, ATP ...
Complex I biogenesis
GeneRatio
Count
padj
C
Relative expression of FOXM1
Hela
ns
Siha
ns
scramble
HN KD
scramble
HN KD
D
R2=0.685
P=0.085
H229
MS751
Hela
Caski
Siha
Relative expression of FOXM1-S
Relative expression of HNRNPC

HNRNPC

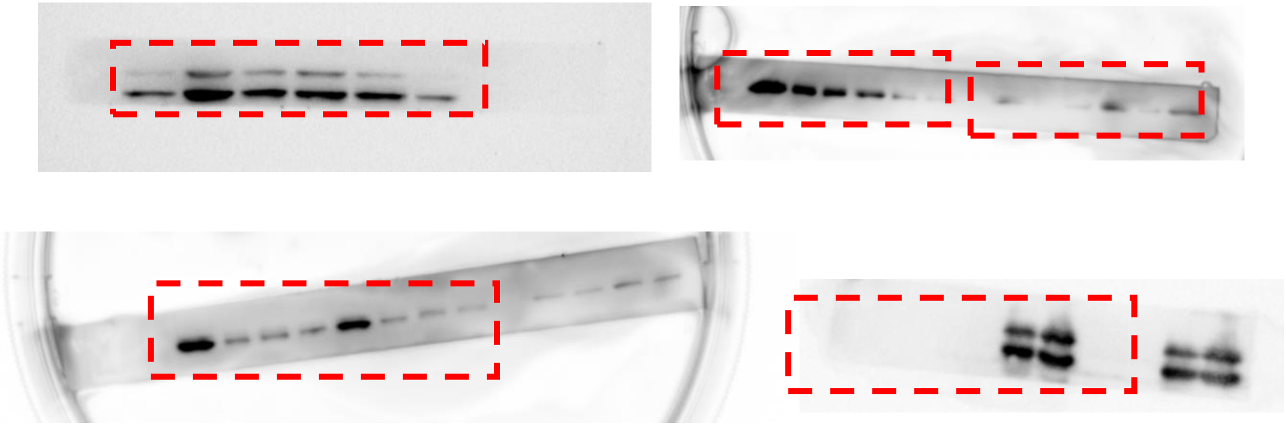

GAPDH

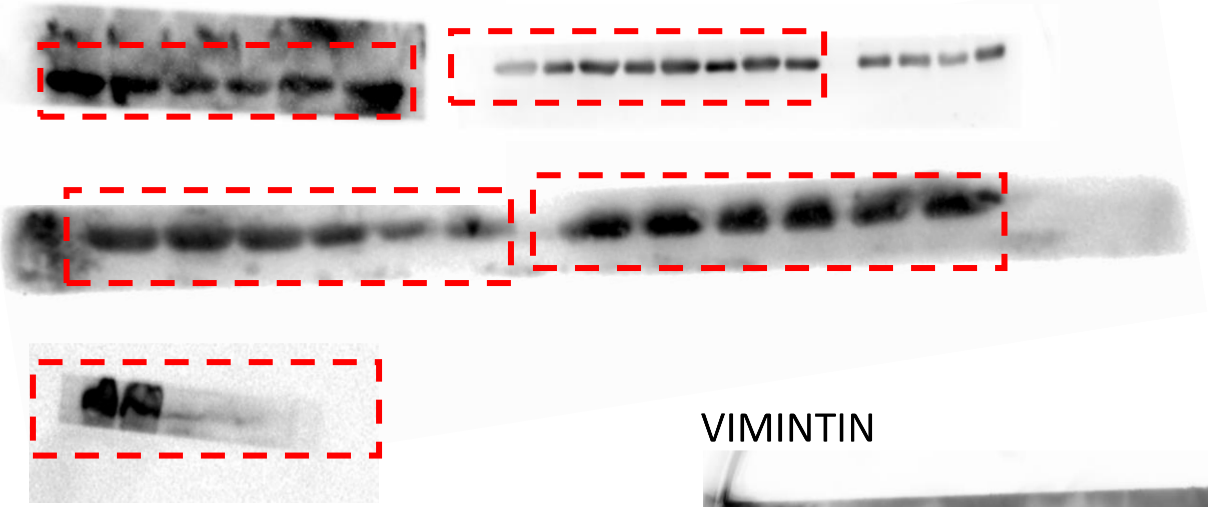

VIMINTIN

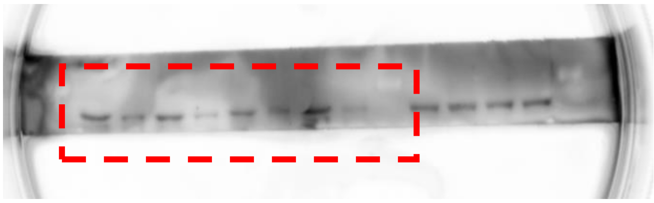

LaminB

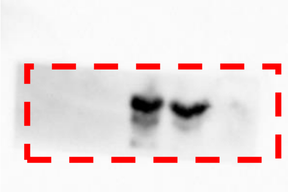

MMP2

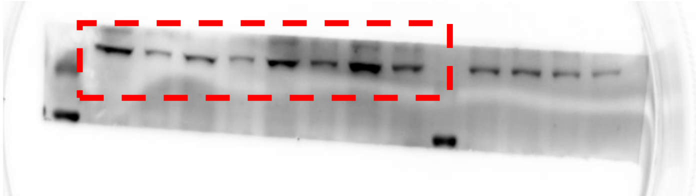

snail

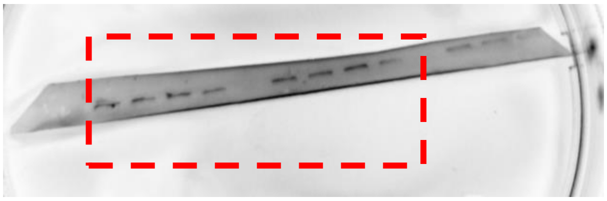

MMP9

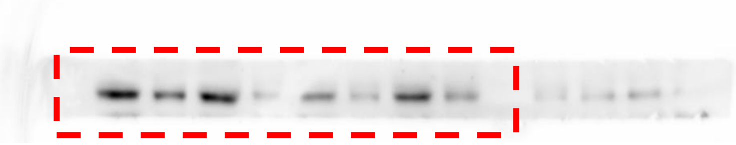

E-CAD

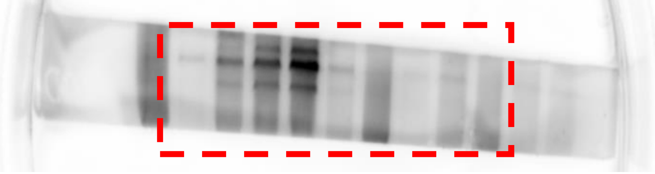

Supplementary figure legends

Figure S1. HNRNPC Expression in CCa Tissues and Cells: an Unfavorable Risk Factor for PFS.

A: TCGA data mining depicts expression levels of 25 m6A-related genes in cervical cancer and normal cervix. B: Comparison across 7 analyses in Oncomine atlas reveals higher expression of HNRNPC in cervical cancer compared to normal cervix (P=0.017). C: RNA expression levels of HNRNPC in cervical cancer cell lines and immortalized cervical cells. D: Protein expressions of HNRNPC in cervical cancer cell lines and immortalized cervical cells. E: Left: HNRNPC staining in cervical cancer (positive staining) and adjacent normal cervix (negative staining) in the same patient; Right: HNRNPC staining in cervical cancer (positive staining) and adjacent CIN lesion (weak staining) in another patient. F: Composition ratio histogram of lymph node status in the two cohorts. G-H: PFS curve in the two cohorts.

Figure S2. Footpad-Popliteal Lymphatic Metastasis Model Indicates Enhanced Metastasis to Regional Lymph Nodes by HNRNPC in Siha Cells In Vivo.

A-B: Immunohistochemistry (IHC) of pan-CK staining in popliteal lymph nodes of mice injected with Siha scramble cells. C-D: Hematoxylin and eosin (HE) staining of popliteal lymph nodes in mice injected with Siha scramble cells. E-F: IHC of pan-CK staining in popliteal lymph nodes of mice injected with HNRNPC knocked-down Siha cells. G-H: HE staining of popliteal lymph nodes in mice injected with HNRNPC knocked-down Siha cells.

Figure S3. Nuclear Localization of HNRNPC Protein in CCa Cells and tissues.

A: Immunofluorescence staining reveal nuclear localization of HNRNPC (red), while control staining of HMGCS1 (green) localizes in both cell membrane and nucleus. B: Immunohistochemistry (IHC) staining demonstrates nuclear localization of HNRNPC in cervical squamous cell tumors and adenocarcinomas. C: Nuclear-cytoplasmic separation analyses confirm HNRNPC expression in the nucleus of Hela and Siha cells.

Figure S4. Downstream Analysis of HNRNPC.

A: Functional enrichment analysis of KEGG in Hela and Siha cells. B: Functional enrichment analysis of Reactome in Hela and Siha cells. C: PCR validation of FOXM1 total RNA levels after HNRNPC knockdown. D: Regression analysis and fitting curve depicting the relationship between HNRNPC and FOXM1-S expressions in cervical cancer cell lines.

Figure S5. Original Western Blot figure

Table S1. Patients information

Ten CCa patients with tumor smaller than 2 cm

ID	age	pathology	grade	LN	FIGO stage	tumor size(cm)
1	59	adenocarcinoma	medium	negative	IIA1	1.5
2	48	squamous cell carcinoma	high	negative	IB1	1.8
3	38	squamous cell carcinoma	low	negative	IB1	1.2
4	61	squamous cell carcinoma	medium	negative	IB1	1
5	59	squamous cell carcinoma	medium	negative	IB1	1.3
6	58	squamous cell carcinoma	high	negative	IB1	1.8
7	47	squamous cell carcinoma	medium	negative	IIA1	1.5
8	61	squamous cell carcinoma	high	negative	IB1	1.3
9	47	squamous cell carcinoma	high	negative	IB1	1.5
10	40	squamous cell carcinoma	medium	negative	IB1	1.8

Seventeen patients with benign uterus or ovarian disease who underwent total hysterectomy

ID	age	diagnosis
1	57	ovarian cystadenoma
2	59	leiomyoma
3	50	leiomyoma
4	51	leiomyoma
5	45	leiomyoma
6	47	ovarian cystadenoma
7	48	leiomyoma
8	53	ovarian cystadenoma
9	42	leiomyoma
10	49	leiomyoma
11	45	leiomyoma
12	58	leiomyoma
13	46	leiomyoma
14	48	leiomyoma
15	49	leiomyoma
16	48	leiomyoma
17	45	leiomyoma
18	48	ovarian cystadenoma
19	72	leiomyoma
20	48	leiomyoma
21	49	leiomyoma

Sixty-one CCa patients (PCR test)

ID	age	pathology	grade	LN	FIGO stage
1	59	adenocarcinoma	low	positive	IIIC1
2	64	squamous cell carcinoma	high	negative	IB3
3	44	squamous cell carcinoma	high	negative	IB1
4	41	squamous cell carcinoma	high	positive	IIIC1
5	61	squamous cell carcinoma	low	negative	IB1
6	62	squamous cell carcinoma	high	positive	IIIC1
7	55	adenocarcinoma	high	positive	IIIC1
8	21	squamous cell carcinoma	high	negative	IB2
9	36	squamous cell carcinoma	medium	negative	IB3
10	36	squamous cell carcinoma	medium	positive	IIIC1
11	62	squamous cell carcinoma	high	negative	IIA1
12	54	squamous cell carcinoma	high	positive	IIIC1

13	49	squamous cell carcinoma	medium	negative	IIA2
14	31	squamous cell carcinoma	high	positive	IIIC1
15	67	squamous cell carcinoma	high	positive	IIIC1
16	39	adenocarcinoma	high	negative	IB1
17	55	squamous cell carcinoma	medium	negative	IB3
18	37	squamous cell carcinoma	high	positive	IIIC1
19	36	squamous cell carcinoma	high	positive	IIIC1
20	48	squamous cell carcinoma	medium	positive	IIIC1
21	64	squamous cell carcinoma	medium	negative	IB1
22	58	adenocarcinoma	medium	negative	IB2
23	50	adenocarcinoma	high	negative	IB2
24	47	squamous cell carcinoma	medium	negative	IIA1
25	40	adenocarcinoma	high	positive	IIIC1
26	60	squamous cell carcinoma	medium	positive	IIIC1
27	38	squamous cell carcinoma	medium	negative	IB2
28	50	squamous cell carcinoma	high	negative	IB2
29	55	squamous cell carcinoma	medium	negative	IB2
30	49	squamous cell carcinoma	medium	positive	IIIC1
31	55	adenocarcinoma	high	positive	IIIC1
32	60	squamous cell carcinoma	medium	positive	IIIC1
33	55	squamous cell carcinoma	medium	negative	IIA1
34	26	squamous cell carcinoma	high	negative	IIA1
35	59	squamous cell carcinoma	medium	negative	IIA2
36	46	squamous cell carcinoma	high	positive	IIIC1
37	57	squamous cell carcinoma	high	negative	IB3
38	59	squamous cell carcinoma	medium	negative	IIA2
39	31	adenocarcinoma	high	positive	IIIC1
40	56	adenocarcinoma	medium	negative	IB2
41	51	squamous cell carcinoma	high	positive	IIIC1
42	57	squamous cell carcinoma	high	negative	IB1
43	68	squamous cell carcinoma	medium	negative	IB3
44	57	adenocarcinoma	medium	negative	IB2
45	46	squamous cell carcinoma	medium	negative	IB3
46	38	adenocarcinoma	high	negative	IIA1
47	53	squamous cell carcinoma	high	negative	IIA1
48	62	squamous cell carcinoma	high	negative	IB2
49	66	squamous cell carcinoma	high	positive	IIIC1
50	56	squamous cell carcinoma	high	negative	IB1
51	63	adenocarcinoma	high	negative	IB2
52	49	squamous cell carcinoma	high	positive	IIIC1
53	58	squamous cell carcinoma	high	negative	IB2
54	54	squamous cell carcinoma	medium	negative	IIA1
55	39	squamous cell carcinoma	high	positive	IIIC1
56	40	squamous cell carcinoma	medium	negative	IIA1
57	38	squamous cell carcinoma	medium	negative	IB2
58	56	adenocarcinoma	medium	positive	IIIC1
59	41	squamous cell carcinoma	high	negative	IB1
60	35	squamous cell carcinoma	high	positive	IIIC1
61	65	squamous cell carcinoma	medium	negative	IB2

Table S2. The sequences of primers in PCR.

GAPDH-F	GGAAGCTTGTCATCAATGGAAATC
GAPDH-R	TGATGACCCTTTTGGCTCCC
HNRNPC-F	GGAGATGTACGGGTCAGTAACA
HNRNPC-R	CCCGAGCAATAGGAGGAGGA
FoxM1 motif1-F	CGGAAGATGAAGCCACTGCT
FoxM1 motif1-R	AATGCGGACTCGCTTGCTAT
FoxM1 motif2-F	TCTTTCTTTGTTTATCAGTGCTGC
FoxM1 motif2-R	TTCCCTCGTCCTGCAGAAGA
FoxM1 motif3-F	CTGCAGGACCAGGGAAAGAG
FoxM1 motif3-R	CCTCCTTGATAGTCTGAACTGGA
FoxM1 motif4-F	TGCAGCCAATCGTTCTCTGA
FoxM1 motif4-R	CCAGTTGATGTTGTCAGGGC
FoxM1-L F	ATAGCAAGCGAGTCCGCATT
FoxM1-L R	AGCAGCACTGATAAACAAAGAAAG
FoxM1-S F	CTACCACGGGTCAGCTCATAC
FoxM1-S R	TCAGCTAGCAGCACCTTTGG

Table S3. RNAi target sequences.

WTAP target sequence	GCUUUGGAGGGCAAGUACATT
HNRNPC target sequence-1	AUUAACAUACUGAAGGAAGGC
HNRNPC target sequence-2	AAUUUGAUCUUAGACAAGCGC

Table S4. Cox regression of clinical-pathology information for overall survival in local cohorts

Cox regression in cohort 1

	univariate regression			multivariate regression		
	upper 95% CI	lower 95% CI	P value	upper 95%	lower 95% CI	P value
HNRNPC	2.06	654.887	**0.014***	1.977	54.124	**0.006***
Lymph cell infiltration	0.142	7.045	0.999			
Age	0.968	1.145	0.234			
Stage	1.219	8198.927	**0.041***			0.363
Pathology	1.662	77.534	**0.013***	1.448	9.558	**0.006***
Grade	0.057	2.304	0.282			
Tumor size	1.183	4.412	**0.014***	1.316	3.173	**0.001***
LVSI	0.664	5.222	0.237			
Lymph node status	1.009	51.493	**0.049***			0.242
Myometrial invasion	0.033	3.092	0.326			
Parametrial infiltration	0.012	2.726	0.218			
Vaginal stump involvement	0	1.708	0.075			0.896

Cox regression in Cohort 2

	univariate regression			multivariate regression		
	upper 95% CI	lower 95% CI	P value	upper 95% CI	lower 95% CI	P value
HNRNPC	2.749	596.834	**0.007***	1.678	32.905	**0.008***
Lymph cell infiltration	0.029	0.482	**0.003***	0.12	0.826	**0.019***
Age	1.004	1.267	**0.043***	1.032	1.173	**0.003***
BMI	0.097	18489.723	0.228			
Stage	0.001	0.528	**0.018***			0.517
Pathology	0.006	0.996	0.05			
Grade	0.085	1.286	0.11			
Tumor size	0.646	1.758	0.804			
LVSI	0.14	2.021	0.335			
Lymph node status	2.445	134223	**0.023***			0.573
Myometrial invasion	0.938	24.449	0.06			
Endometrial involvement	0.015	0.648	**0.016***			0.993
Parametrial infiltration	0.106	10.126	0.976			
Vaginal stump involvement	1.036	52.88	**0.046***			0.971

*:P $<$0.05

Supplementary table legends

Supplementary table 1: Patients information

Information of age, pathology, grade, lymph node status, and FIGO stage of patients were included in supplementary table 1. The diagnosis of the patients who underwent total hysterectomy were also included.

Footnote and abbreviations:

LN: lymph node status; FIGO: International Federation of Gynecology and Obstetrics

Supplementary table 2: The sequences of primers in PCR.

Supplementary table 3: RNAi target sequences.

Supplementary table 4: Cox regression of clinical-pathology information for overall survival in local cohorts

Footnote and abbreviations:

CI: confidential interval; *: $P < 0.05$
